# Evaluation of an *in vitro* fibre fermentation method using feline faecal inocula: inter-individual variation

**DOI:** 10.1017/jns.2017.21

**Published:** 2017-05-24

**Authors:** Guido Bosch, Lisa Heesen, Karine de Melo Santos, John W. Cone, Wilbert F. Pellikaan, Wouter H. Hendriks

**Affiliations:** 1 Animal Nutrition Group, Wageningen University, PO Box 338, 6700 AH Wageningen, The Netherlands; 2 Faculty of Veterinary Medicine, Utrecht University, PO Box 80.151, 3508 TD Utrecht, The Netherlands

**Keywords:** Cats, Fibre fermentability, *In vitro* methodology, Gas production, SCFA, CP, citrus pectin, FOS, fructo-oligosaccharide, GG, guar gum, *R*_max_, maximum rate of gas production, SBP, molassed sugar beet pulp, *T*_max_, time at which *R*_max_ occurred, WM, wheat middlings

## Abstract

The present study aimed to evaluate the inter-individual variability in fermentation of standard fibrous substrates by faecal inocula from ten healthy adult female cats. Substrates were citrus pectin (CP), fructo-oligosaccharides (FOS), guar gum (GG), sugar beet pulp (SBP) and wheat middlings (WM). Each substrate was incubated with faecal inoculum from each cat. Gas production was measured continuously during the 48 h incubation and SCFA and organic matter disappearance (only SBP and WM) after incubation. Out of ten cats, nine produced faeces on the days of inoculum preparation. The substrates contrasted in terms of fermentation parameters measured. The inter-individual variability was in general lower for the more simple and pure substrates (CP, FOS, GG) than for the more complex substrates containing mixtures of fibres (SBP, WM). Furthermore, for total SCFA and gas produced, inter-individual variability was lower than for proportions of butyrate and of branched-chain fatty acids and for the parameters of gas production kinetics. It is concluded that the variability in *in vitro* fermentation parameters is associated with the complexity of fibrous substrates. The presented data are instrumental for the calculation of number of faecal donors required for precise *in vitro* characterisation of the fermentability of dietary fibres. In addition, the number of faecal donors should be adjusted to the specific fermentation parameter(s) of interest.

The properties of dietary fibres may affect cats’ health, digestive processes and faecal characteristics^(^^1^^)^. It is therefore important to consider the properties of dietary fibres including their potential fermentability by the intestinal microbiota. To characterise the fermentability of fibres, *in vitro* methods simulating intestinal fermentation have been developed. *In vitro* batch culture systems are commonly applied in which the fibrous substrate of interest is incubated with a faecal inoculum from the target animal species. There are, however, methodological aspects that can have an impact on the quality of the results obtained and these are not well described in the literature. For example, the number of faecal donors has been advised to be at least five to control for the effect of inter-individual variation^(^^2^^)^. Most studies have used three faecal donors^(^^3^^)^. However, some only used one^(^^4^^)^. Furthermore, few studies focused on the repeatability and reproducibility of the method, which has been described for our laboratory in a companion article^(^^5^^)^. To gain knowledge on the precision of an *in vitro* method for characterisation of the fermentability of dietary fibres, this study aimed to evaluate the inter-individual variability in fermentation of five standard fibrous substrates by faecal inocula from ten cats.

## Experimental methods

### Substrates

Selected dietary fibres or fibre sources are used in dry and wet cat foods and contrast in chemical composition and anticipated fermentation characteristics, as shown in previous *in vitro* studies^(^^6^^–^^8^^)^. Substrates included citrus pectin (CP; rapidly and highly fermentable, HM Rapid, TIC Gums), fructo-oligosaccharide (FOS; rapidly and highly fermentable; Orafti^®^ IPS, BENEO-Orafti), guar gum (GG; rapidly and highly fermentable, 8/22, TIC Gums), molassed sugar beet pulp (SBP; slowly and highly fermentable; Research Diet Services) and wheat middlings (WM; slowly and moderately fermentable; Research Diet Services).

### Animals, housing and care

A total of nine intact and one neutered female European shorthair cats (3 to 5 years old) with a mean body weight of 3·3 (sd 0·6) kg were used. Cats did not receive any antibiotics for at least 6 months prior to faecal collection. The cats were housed in two groups in rooms with an outside area and individually in a metabolic cage during parts of the day (12.00–13.30 and 16.30–09.30 hours). Details of the group rooms and metabolic cages as well the climate and light schedules have been provided by Van Rooijen *et al.*^(^^9^^)^. Litter trays were present in the cage and contained non-absorbent polyethylene litter (Katkor^®^; Rein Vet Products). On the day of faeces collection the litter and the tray were sterilised with 70 % ethanol. Cats were fed a nutritionally complete (i.e. meeting the FEDIAF standards) commercial dry extruded diet (Perfect Fit In-Home; Mars Petcare) for at least 4 weeks prior to faeces collection. Cats were adapted in 2 weeks to a daily regimen of feeding between 08.30 and 09.30 hours (about 45 % of their daily portion), 12.00 and 13.30 hours (about 10 %) and at 16.30 hours (about 45 %) in their cage and provided food to maintain optimal body weight. In the morning and afternoon, cats went to their group room. Water was available *ad libitum* during group housing and in the metabolic cages. The health status of the animals was monitored daily and cats were weighed weekly. Animal care and experimental procedures were approved by the Animal Care and Use Committee of Wageningen University, Wageningen, the Netherlands.

### Preparation of inoculum and incubation

Within 15 min of defecation, faeces were transferred to sterile 250 ml plastic bottles prefilled with CO_2_ and 250 ml of CO_2_ was immediately added to the bottle to ensure anaerobic storage conditions. The bottle with faeces was closed and transported within 5 min to the analytical laboratory. Faeces from cats were not pooled but processed for each cat. All handling procedures and processing of faeces to the inoculum were carried out under a constant stream of CO_2_. All attached litter particles were manually removed from faeces and faeces were weighed and diluted 1:9 (w/v) in a 39°C anaerobic sterile physiological saline solution (9 g/l NaCl). The diluted mixture was homogenised for 60 s using a hand-blender and filtered through nylon fabric (pore size 40 µm, permeability 30 %; PA 40/30, Nybolt). The filtrate was mixed with a pre-warmed (39°C) N-containing medium^(^^10^^)^ in a 5:84 mixture (v/v) and gently flushed for 5 min with CO_2_. The resulting medium/inoculum mixture was dispensed (89 ml) into pre-warmed and CO_2_-flushed 250 ml serum bottles (Schott) containing 0·5 g of substrate. Inoculated bottles were then immediately attached to fully automated gas production equipment^(^^11^^)^ and gas production was recorded for 48 h. This incubation time is longer than average total tract transit times observed in young adult (36 (sd 14) h) and senior cats (26 (sd 6) h)^(^^12^^)^ with a orocaecal transit time of approximately 5 h^(^^13^^)^. However, 48 h was estimated to be required for characterising the fermentation kinetics of SBP and WM. After 48 h of incubation, fermentation liquids were sampled for the determination of SCFA (i.e. acetate, propionate, butyrate, iso-butyrate, valerate, iso-valerate) concentrations and for organic matter disappearance of SBP and WM. All incubations were done in triplicate. The study was performed in two runs, with maximally five inocula per run.

### Chemical analyses

All substrates were analysed for DM and ash by drying to a constant weight at 103°C and combusting at 550°C, respectively. The SBP and WM were also analysed for crude protein (6·25 × N)^(^^14^^)^, for neutral-detergent fibre (NDF)^(^^15^^,^^16^^)^, and for acid-detergent fibre (ADF) and acid-detergent lignin (ADL)^(^^17^^)^. SCFA analyses of fermentation liquids as well as organic matter disappearance were analysed as described by Bosch *et al.*^(^^18^^)^.

### Calculations and data analyses

Cumulative gas production (ml) and SCFA production (mmol) were expressed per g organic matter. Monophasic models as described by Groot *et al.*^(^^19^^)^ were fitted to the data for cumulative gas production and the maximum rate of gas production (*R*_max_ in ml/(g organic matter h)) and the time at which it occurred (*T*_max_ in h) were calculated^(^^20^^)^. Acetate, propionate and butyrate were expressed as percentage of total SCFA. Similarly, branched-chain proportion was iso-butyrate + iso-valerate on a total SCFA basis. Values of replicates for each substrate–cat combination were averaged and considered as the experimental unit. For each substrate, mean and standard deviation values were calculated and used to compute the CV (sd/mean × 100).

## Results and discussion

**Fig. 1.**
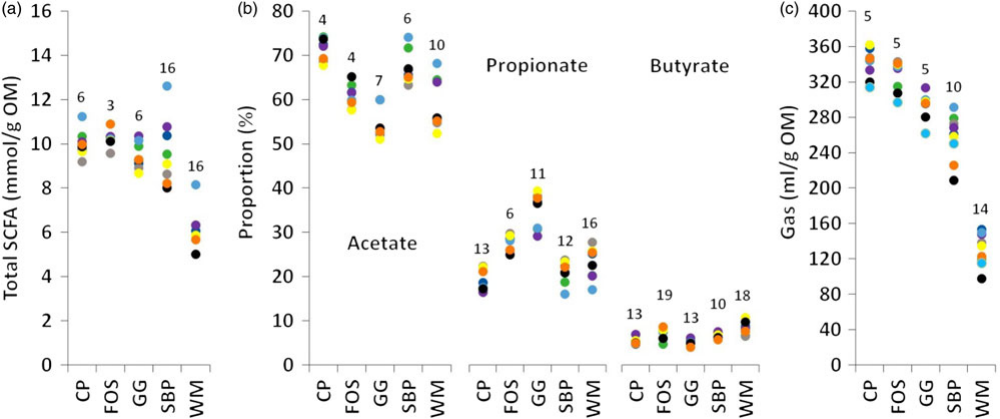
Inter-individual variation in *in vitro* total SCFA production (a), proportions of acetate, propionate and butyrate (b) and gas production (c). Coloured markers represent results from individual cats used as faecal donors (*n* 8 for SCFA and *n* 9 for gas production). CV (%) per substrate is indicated above the markers. OM, organic matter; CP, citrus pectin; FOS, fructo-oligosaccharide; GG, guar gum; SBP, molassed sugar beet pulp; WM, wheat middlings.

All cats remained healthy throughout the study. Of the ten cats, nine produced faeces on the days of inoculum preparation and for one cat the SCFA analysis failed. DM and ash contents (as is) were CP 90·0 and 1·7 %, FOS 96·5 and 0·0 %, GG 89·4 and 0·7 %, SBP 89·3 and 6·3 % and WM 88·4 and 7·2 %, respectively. Crude protein, NDF, ADF and ADL contents were, respectively, 9·2, 32·2, 16·6 and 1·0 % for SBP and 15·7, 42·3, 14·0 and 3·9 % for WM. The substrates contrasted in terms of fermentation parameters measured, as anticipated (Fig. 1 and Table 1). Furthermore, the ranking of substrates (i.e. relative accuracy^(^^3^^)^) was consistent among cats (based on Spearman's rank correlations; results not shown).

**Table 1.** Mean values and inter-individual variability of *in vitro* fermentation parameters for fibrous substrates using feline faecal inocula (*n* 9 donor cats unless otherwise indicated)

Parameter		Substrate				
		CP	FOS	GG	SBP	WM
BCP (% of total SCFA production)*	Mean	2·3	2·3	2·9	2·8	5·7
	CV (%)	10	9	11	22	19
*R*_max_ (ml/(g OM h))	Mean	87	85	51	–	–
	CV (%)	15	11	32	–	–
*T*_max_ (h)	Mean	3·8	4·1	5·0	–	–
	CV (%)	12	17	23	–	–
OMD (% of initial weight)	Mean	–	–	–	86	49
	CV (%)	–	–	–	12	12

CP, citrus pectin; FOS, fructo-oligosaccharide; GG, guar gum; SBP, molassed sugar beet pulp; WM, wheat middlings; BCP, branched-chain proportion; *R*_max_, maximum rate of gas production; OM, organic matter; *T*_max_, time at which *R*_max_ occurred; OMD, organic matter disappearance.

* *n* 8.

Total SCFA production from CP, FOS, GG and SBP was similar and greater than the production from WM. Inter-individual CV of total SCFA was larger for SBP and WM than for CP, FOS and GG (Fig. 1(a)). Few other studies focused on inter-individual variation in *in vitro* fermentation of fibrous substrates using faecal inocula. The CV for *in vitro* total SCFA production after 24 h of incubation with faecal inocula from healthy human volunteers ranged from 9 % for GG to 15 % for wheat bran (*n* 6 individuals)^(^^21^^)^ and was 11 % for soyabean fibre (*n* 4)^(^^22^^)^. In a large study with eight laboratories and number of faecal inocula from healthy human volunteers ranging from 4 to 7, inter-individual CV of total SCFA production after 24 h of incubation was larger for potato starch being pregelatinised (median 18, range 6–29 %), raw (22, 13–44 %) and retrograded (23, 15–55 %) and for glassy pea starch (22, 18–39 %)^(^^2^^)^. The SCFA composition differed between substrates with the greatest acetate and propionate proportions for CP and GG, respectively. The CV of the proportion of acetate was smaller than for propionate and butyrate (Fig. 1(b)), which was also found for soyabean fibre fermented by human faecal inocula^(^^22^^)^, i.e. respectively 8, 16 and 45 %. The variation in absolute values was in particular small for butyrate in the present study. Inter-individual CV of gas production was like that for total SCFA: larger for SBP and WM than for CP, FOS and GG (Fig. 1(c)). McBurney & Thompson^(^^21^^)^ reported larger CV for gas production ranging from 9 % for kidney beans to 23 % for GG. Incubation with WM resulted in the largest branched-chain proportion (Table 1), which relates to the relatively high protein content, and was also observed in an *in vitro* study with faecal inocula from dogs^(^^23^^)^. The inter-individual variation in branched-chain proportion was in particular large for SBP and WM. Fitting of the model for gas production was not possible for SBP and WM. The *R*_max_ and *T*_max_ were similar for CP and FOS. For GG, *R*_max_ was lower than for CP and FOS and inter-individual variation in *R*_max_ and *T*_max_ was in particular large for GG. Finally, WM had a lower organic matter disappearance than SBP after 48 h of incubation, but both substrates showed similar inter-individual variability.

The observed fibre source-dependent inter-individual variation in various fermentation parameters probably relates to differences in specific microbial taxa observed in cat faeces^(^^24^^)^. In healthy humans, variation in microbial taxa was also noted but the functionality of the microbiome (i.e. metabolic pathways) remained fairly stable among individuals^(^^25^^)^. Degradation of more complex and mixtures of non-digestible carbohydrates would require a larger array of microbial enzymes than that required for the degradation of relatively simple and pure carbohydrates. Differences in faecal microbial composition and its functional capacities among individual cats may therefore become more pronounced with the more complex fibrous substrates (SBP and WM) relative to the more simple and pure ones (CP, FOS and GG). The variability associated with the complexity of fibrous substrates presented in this study is instrumental for the calculation of the number of faecal donors required for precise *in vitro* characterisation of the fermentability of dietary fibres. In addition, the number of faecal donors should be adjusted to the specific fermentation parameters of interest.
